# Subconjunctival Eyelash Removal With a 27-Gauge Needle: A Novel Minimally Invasive Technique in Five Cases

**DOI:** 10.7759/cureus.102029

**Published:** 2026-01-21

**Authors:** Tatsuya Mimura, Daisuke Hasegawa, Yui Nishijima, Hidetaka Noma, Ryuta Kimoto, Kenichiro Yamazaki

**Affiliations:** 1 Ophthalmology, Tsurumi University School of Dental Medicine, Yokohama, JPN; 2 Ophthalmology, Ibaraki Medical Center, Tokyo Medical University, Inashiki-gun, JPN; 3 Ophthalmology, Omiya Nanasato Eye Institute, Saitama, JPN

**Keywords:** 27-gauge needle, cilium migration, minimally invasive extraction, slit-lamp guidance, subconjunctival eyelash, subconjunctival foreign body

## Abstract

Subconjunctival migration of eyelashes is an extremely rare phenomenon, and most reported cases are asymptomatic and managed conservatively. In this report, we describe five cases in which subconjunctival eyelashes were successfully and safely removed using a 27-gauge needle. Subconjunctival cilia were identified in five eyes of five female patients aged 42, 52, 53, 78, and 89 years. Under topical anesthesia with benoxinate hydrochloride and slit-lamp observation, a 27-gauge needle was used to puncture the conjunctiva beneath the eyelash, gently guiding its central portion toward the conjunctival surface, after which the eyelash was extracted using fine forceps. In all cases, the procedure was completed within one minute and was associated with minimal intraoperative and postoperative hemorrhage. To our knowledge, this is the first report demonstrating that subconjunctival eyelashes can be rapidly and effectively removed using a 27-gauge needle. This minimally invasive and simple technique can be performed in an outpatient setting without the need for an operating room and may represent a practical and effective option for future clinical management.

## Introduction

Subconjunctival migration of eyelashes is an extremely rare phenomenon, and most reported cases remain asymptomatic and clinically quiescent at presentation. Foreign bodies in the subconjunctival space have occasionally been described following ocular trauma, ophthalmic surgery, or procedures such as sub-Tenon’s anesthesia, with eyelashes identified as the causative material in a limited number of reports [1, 2, 3]. The proposed mechanisms for eyelash migration into the subconjunctival space include direct penetration following minor trauma or surgery, inadvertent introduction during periocular procedures, and gradual migration through microdefects in the conjunctiva driven by blinking or eye movements.

In previously published cases, removal of such foreign bodies was often performed using relatively invasive approaches, including conjunctival incision under local or general anesthesia, forceps extraction in the operating room, or procedures requiring surgical exposure of the subconjunctival space. Nevertheless, when the foreign material is a single, asymptomatic eyelash-often barely visible and clinically benign-there is a clear need for a simpler, less invasive method of extraction.

In this report, we present five cases of subconjunctival eyelashes that were asymptomatic and successfully removed under slit-lamp guidance using a 27-gauge needle in an outpatient setting. This technique is quick, simple, and does not require specialized equipment or surgical facilities, making it safe to perform under topical anesthesia in a standard clinical environment.

This study aims to describe the clinical utility of this novel extraction method for a rare condition and to provide a practical approach that may be applicable in similar future cases.

## Case presentation

This study is a case series based on anonymized clinical data obtained during routine medical care. As such, it was deemed exempt from institutional review board (IRB) approval. The study was conducted in accordance with the principles outlined in the Declaration of Helsinki (2013 revision) and relevant national and institutional ethical guidelines. Written or verbal informed consent was obtained from all patients for the publication of their clinical information and accompanying images for academic purposes.

Case one

**Figure 1:**
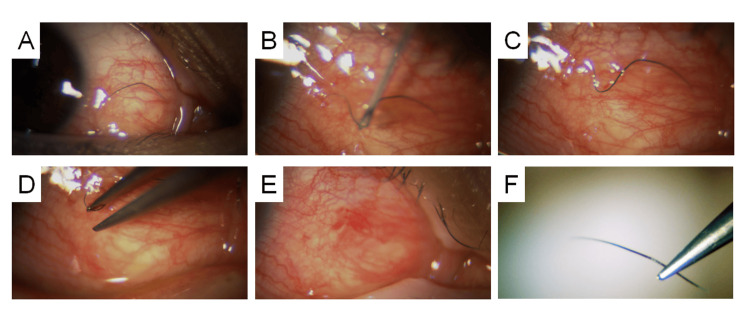
A 42-Year-Old Female. (A) Subconjunctival eyelash located on the nasal side of the right eye. (B) A 27-gauge needle is inserted beneath the subconjunctival eyelash to guide its central portion toward the conjunctival surface. (C) The central part of the eyelash is exposed on the conjunctival surface. (D) The protruding portion is grasped with forceps and extracted. (E) Mild subconjunctival hemorrhage noted postoperatively. (F) Extracted eyelash.

A 42-year-old woman presented with awareness of a black foreign body on the surface of her right eye for two days. Slit-lamp examination revealed a single eyelash embedded in the nasal subconjunctival space of the right eye. Fluorescein staining was negative, and there was no evidence of conjunctival epithelial damage. At the patient’s request, the eyelash was removed under topical anesthesia using a 27-gauge needle to puncture the conjunctiva and guide the central portion of the eyelash to the surface, where it was grasped and extracted with forceps. Mild subconjunctival hemorrhage was noted postoperatively (Figure 1). A single drop of 1.5% levofloxacin ophthalmic solution was administered immediately after the procedure, and no further topical treatment was required.

Case two

**Figure 2:**
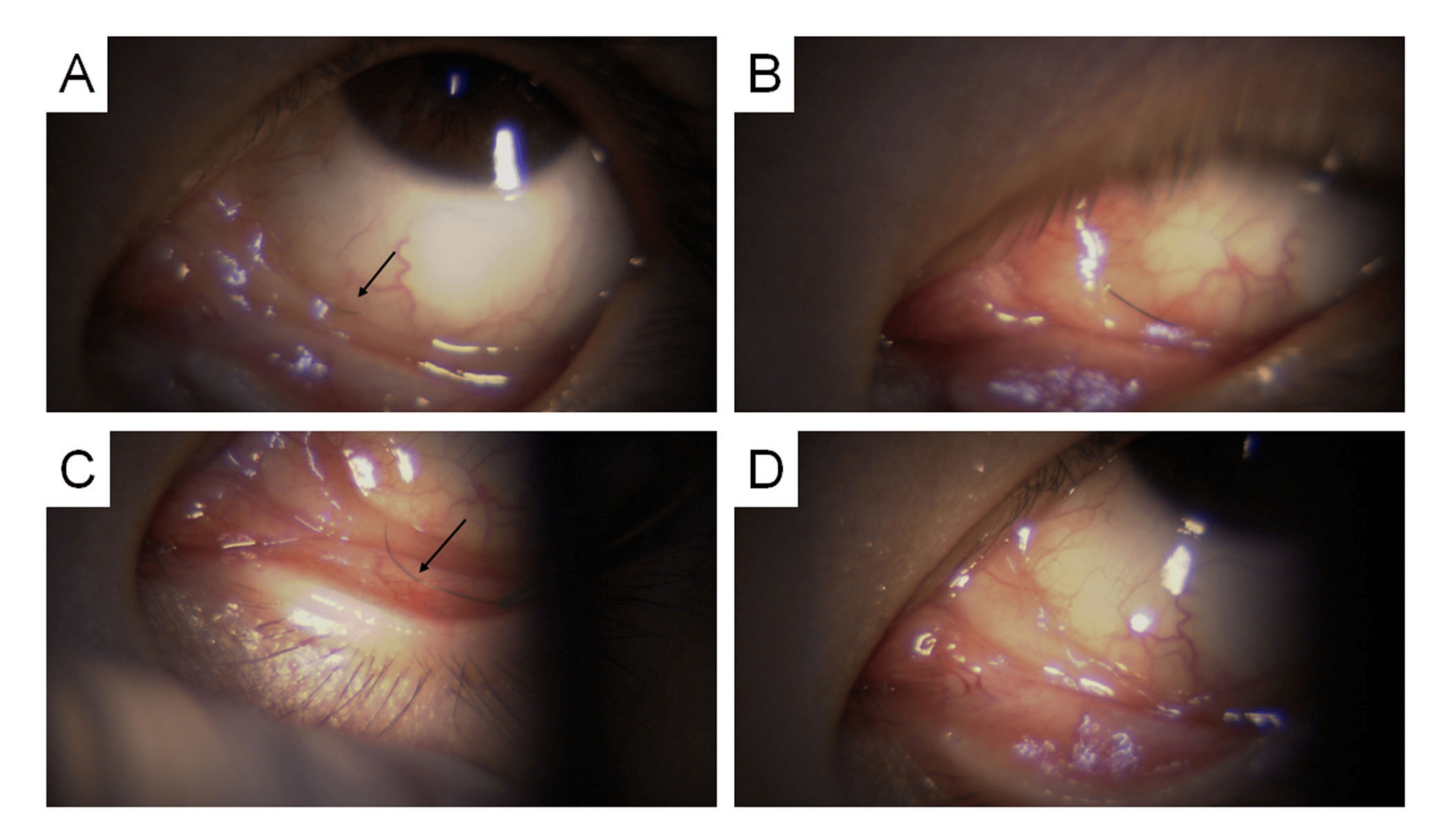
A 52-Year-Old Female. (A) Subconjunctival eyelash (black arrow) migrated from the inferonasal quadrant toward the fornix of the left eye. (B) The eyelash after its central portion was lifted onto the conjunctival surface using a 27-gauge needle. (C) The eyelash (black arrow) is being extracted upward from the lower conjunctiva with forceps. (D) No subconjunctival hemorrhage or visible wound was observed after removal. The slit-lamp microscope used in this case required manual pressing of the shutter button; therefore, intraoperative photographs during needle or forceps manipulation could not be obtained.

A 52-year-old woman presented with a three-day history of foreign body sensation and redness in the left eye. Slit-lamp examination revealed a subconjunctival eyelash in the inferior nasal quadrant. The patient also exhibited a slightly deepened nasal conjunctival fornix and conjunctivochalasis. Under topical anesthesia, a 27-gauge needle was used to puncture the conjunctiva from beneath the eyelash, guiding it to the surface, followed by extraction with forceps. Mild subconjunctival hemorrhage was observed (Figure 2). A single drop of 1.5% levofloxacin was instilled postoperatively; no further treatment was needed.

Case three

**Figure 3:**
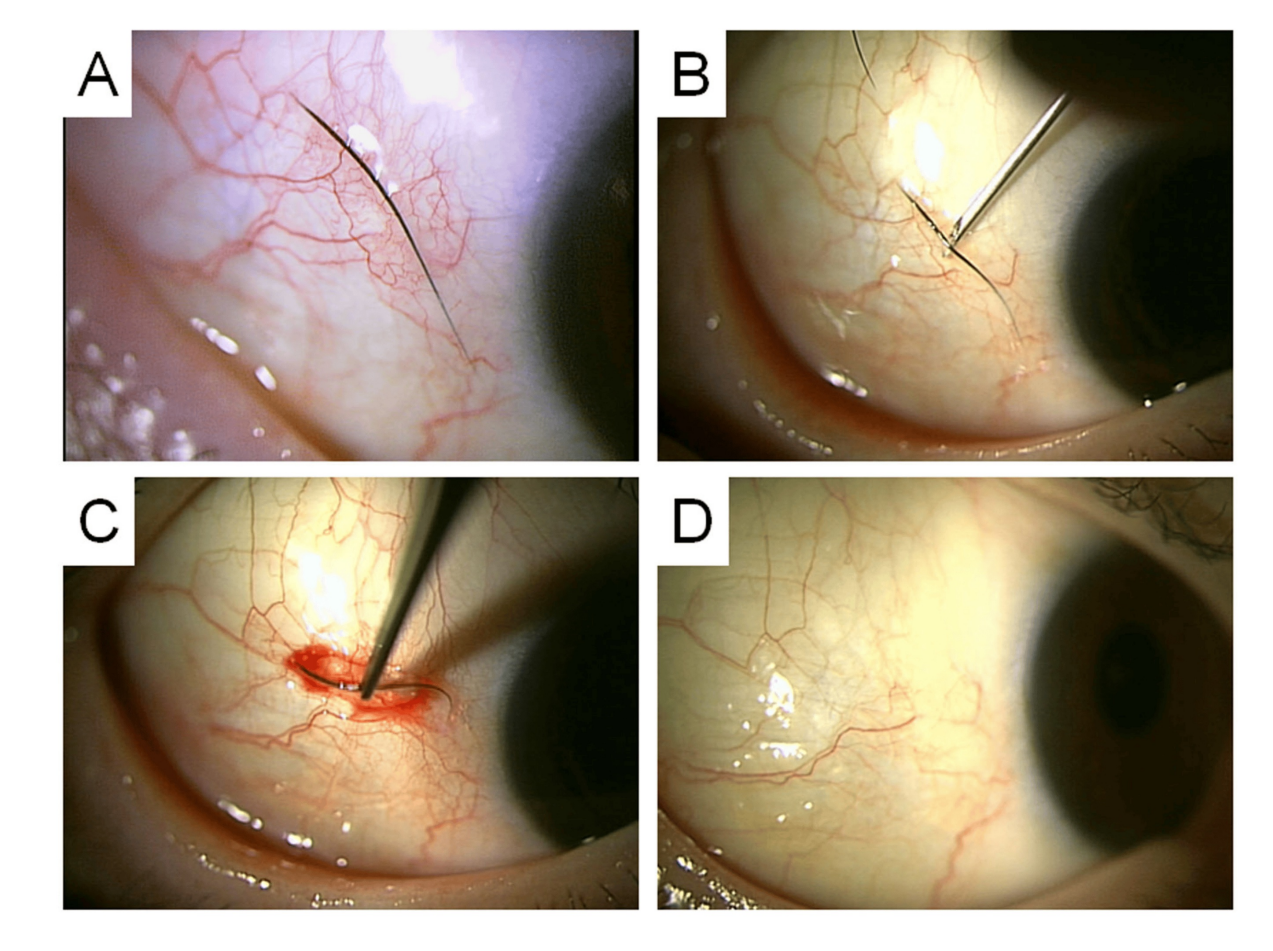
A 53-Year-Old Female. (A) Subconjunctival eyelash on the temporal side of the right eye. (B) A 27-gauge needle is inserted beneath the eyelash to guide its central part to the surface. (C) The exposed portion is grasped with forceps and removed. Mild subconjunctival hemorrhage is observed during the procedure. (D) Conjunctival appearance at 4 weeks post-extraction shows no hemorrhage or congestion.

A 53-year-old woman with no significant systemic medical history and mild myopia (corrected only while driving) presented with a one-week history of an eyelash that appeared to be stuck in the conjunctiva of her right eye. She had also noticed bilateral blurring of vision in the evenings over the past six months. Slit-lamp examination confirmed a subconjunctival eyelash on the temporal side of the right eye. Attempted extraction with forceps was unsuccessful due to the eyelash being entirely beneath the conjunctiva. A 27-gauge needle was then used to puncture the conjunctiva and elevate the central portion of the eyelash to the surface, allowing for successful removal with forceps. Mild subconjunctival hemorrhage was observed during the procedure (Figure 3). A single drop of 1.5% levofloxacin was administered immediately postoperatively. No further treatment was required. At one-week follow-up, the surgical site had healed well, with no residual hemorrhage (Figure 3).

Case four

**Figure 4:**
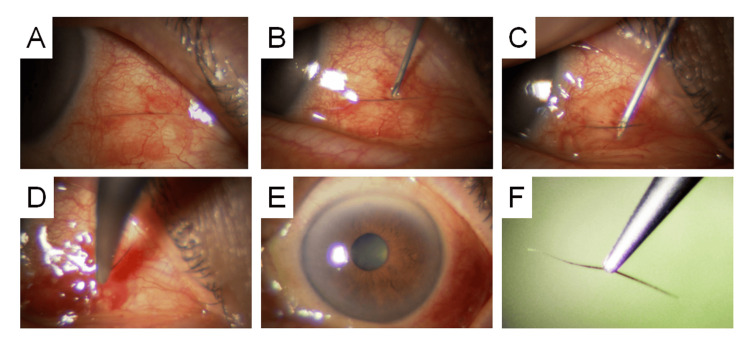
A 78-Year-Old Female with a Subconjunctivally Embedded Eyelash in the Left Eye. (A) An eyelash embedded beneath the temporal bulbar conjunctiva. (B) A 27-gauge needle is used to puncture the conjunctiva and insert the tip beneath the eyelash. (C) The needle is positioned vertically to incise the conjunctiva from the inner side outward, allowing the eyelash to be guided to the surface. (D) The exposed central portion of the eyelash is grasped with forceps and extracted. (E) A mild subconjunctival hemorrhage was observed immediately after the procedure. (F) The extracted eyelash.

A 78-year-old woman presented with a complaint of a persistent eyelash adhered to the conjunctiva of her left eye for several days. She reported no associated pain or discomfort. Slit-lamp examination revealed a misdirected eyelash embedded beneath the temporal bulbar conjunctiva of the left eye. Under topical anesthesia, a 27-gauge needle was used to puncture the conjunctiva beneath the embedded eyelash and gently dislodge it toward the conjunctival surface, after which it was extracted using forceps. Postoperative subconjunctival hemorrhage was minimal (Figure 4). A single dose of 1.5% levofloxacin ophthalmic solution was instilled immediately after the procedure, and no additional topical treatment was administered thereafter.

Case five

**Figure 5:**
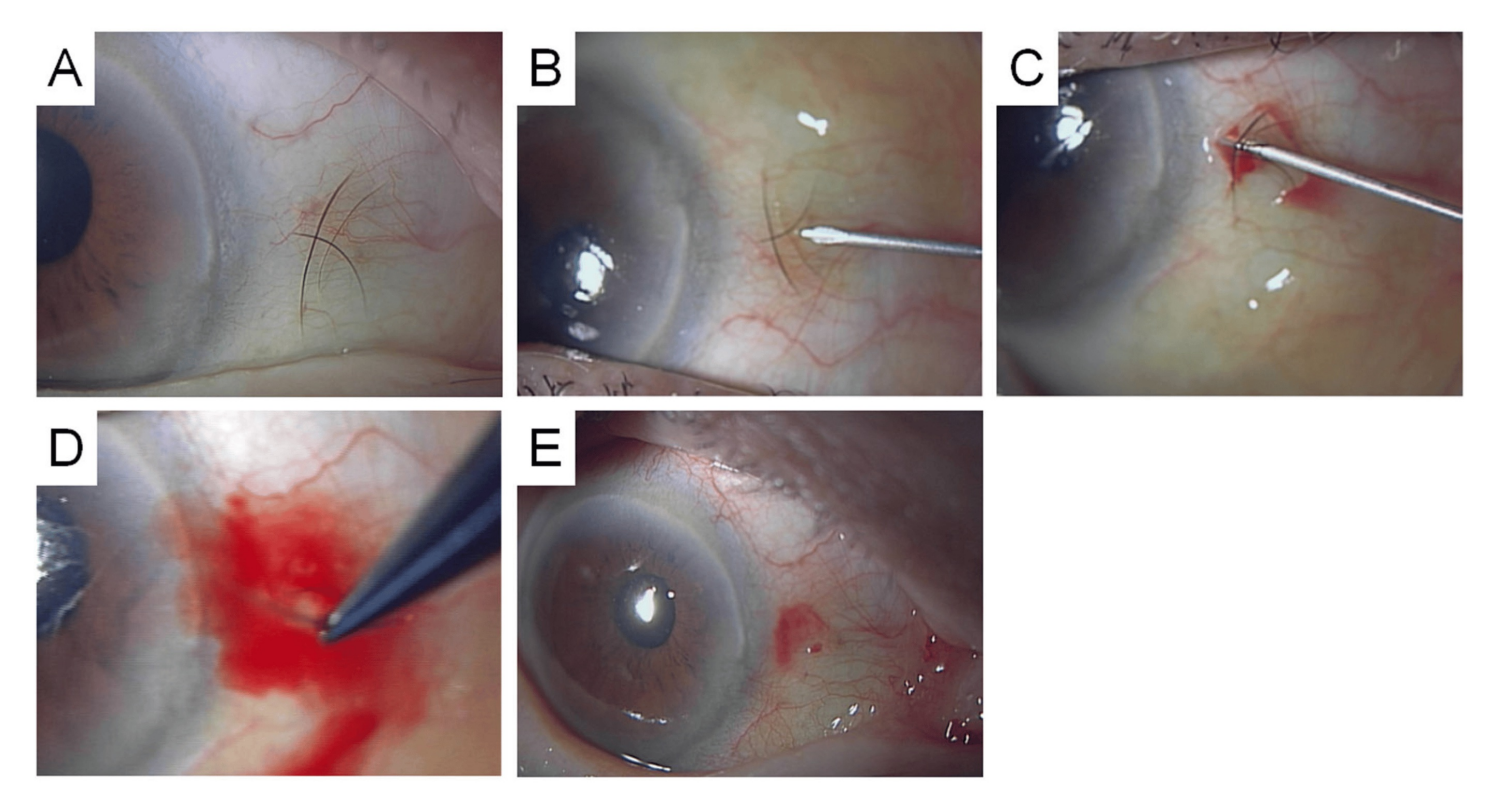
An 89-Year-Old Female. (A) Three subconjunctival eyelashes located on the nasal side of the right eye. (B) A 27-gauge needle is inserted beneath all three lashes. (C) The needle is rotated 90 degrees to incise the conjunctiva vertically from the inside, simultaneously guiding the lashes to the surface. (D) All three lashes are grasped and extracted with forceps. (E) Mild subconjunctival hemorrhage was noted and controlled by compression with a cotton swab immediately after extraction.

An 89-year-old woman, who had undergone bilateral cataract surgery 12 months prior and was undergoing regular follow-up every two to three months, was found to have three eyelashes embedded in the nasal subconjunctival space of her right eye during a routine examination. She reported a mild foreign body sensation. Under topical anesthesia, a 27-gauge needle was used to puncture the conjunctiva beneath the embedded lashes, guiding all three lashes to the surface, where they were extracted simultaneously with forceps. Mild subconjunctival hemorrhage was noted following the procedure (Figure 5). A single postoperative drop of 1.5% levofloxacin was administered, and no additional topical treatment was necessary.

Summary of patient characteristics

Table 1 summarizes the clinical backgrounds of the five cases. All patients were female. Two patients reported a foreign body sensation during blinking, while the other three were asymptomatic. Subconjunctival eyelashes were present in the right eye in three cases and in the left eye in two cases. The location of eyelash embedding was nasal in three cases and temporal in two cases.

**Table 1:** Patient Background.

Case No.	Age / Sex	Surgical History	Symptoms	Location of Subconjunctival Eyelash	Postoperative Subconjunctival Hemorrhage
1	42/F	None	None	Right eye, nasal side	Mild
2	52/F	None	Foreign body sensation	Left eye, nasal side	None
3	53/F	None	None	Right eye, temporal side	Mild
4	78/F	None	None	Left eye, temporal side	Mild
5	89/F	Cataract surgery	Foreign body sensation	Right eye, nasal side	Mild

## Discussion

Summary of the present study

In this case series, we presented a simple and minimally invasive technique using a 27-gauge needle for the extraction of asymptomatic subconjunctival eyelashes. To our knowledge, this is one of the few reports to describe a procedure that can be safely performed in an outpatient setting under slit-lamp guidance, demonstrating its rapid and safe execution despite sporadic prior reports of needle-assisted removal.

**Figure 6:**
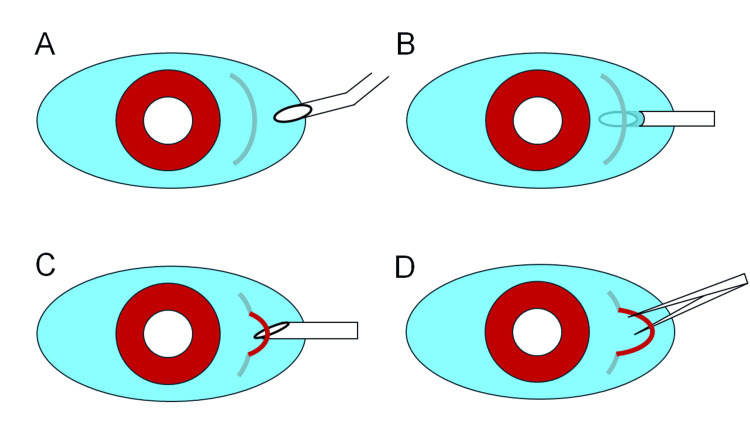
Schematic of the Eyelash Extraction Technique Using a 27-Gauge Needle. (A) The needle is bent 30–45 degrees at its midpoint, bevel-up orientation. (B) The needle tip is gently inserted between the eyelash and sclera from the curved apex side. (C) The needle is rotated 90 degrees, and its lateral edge is used to make a vertical conjunctival incision from inside to surface, exposing the eyelash. (D) The exposed central portion of the eyelash is grasped and removed with forceps. This figure is an original illustration created by the authors for the present study and is not reproduced or adapted from any previously published source.

This method involves several technical tips that facilitate the procedure, as illustrated in Figure 6. First, bending the middle portion of the needle at an angle of 30 to 45 degrees improves maneuverability. When inserting the needle into the subconjunctival space, it should be oriented bevel-up and carefully slid between the eyelash and the scleral surface. Inserting the needle from the convex (curved) side of the eyelash improves the likelihood of successful engagement with the lash.

To expose the eyelash onto the conjunctival surface, the needle should be rotated 90 degrees horizontally, and the lateral edge of the needle tip used to gently incise the conjunctiva from the underside in a vertical lifting motion. This technique allows smooth incision with minimal resistance. Once part of the eyelash emerges from the incision, the central portion of the exposed lash can be grasped with forceps and extracted cleanly.

In cases of bleeding, slight pressure with a cotton swab over the incision site is usually sufficient to control hemorrhage. In all five cases, the procedure was completed within approximately one minute, underscoring its simplicity and efficiency. Notably, no preoperative disinfection or ocular irrigation was required, and in all cases, a single postoperative application of 1.5% levofloxacin ophthalmic solution was sufficient, with no need for additional topical medication.

Patient background and relevant literature comparison

Subconjunctival entrapment of eyelashes is an exceptionally rare clinical phenomenon. George et al. described a single postoperative case, underscoring the uncommon nature of this entity [1]. Similarly, other reports of asymptomatic subconjunctival eyelashes have emphasized their rarity [3]. In most reported cases, a conservative, observational approach was chosen, and active intervention, such as extraction, as in the present series, is seldom documented.

In our series, all five patients were female, and in three out of five eyes, the eyelash was embedded on the nasal side. This apparent gender predisposition may relate to cosmetic practices and hormonal influences. Cosmetic activities predominantly performed by women, such as mascara application, eyelash extensions, and use of eyelash curlers, may mechanically weaken the lashes, increasing the risk of breakage or detachment and subsequent subconjunctival migration. Indeed, eyelash follicles are reported to be particularly sensitive to external cosmetic factors [4]. Moreover, female sex hormones-estrogen and progesterone-are known to affect hair metabolism and immune modulation, potentially influencing micro‐wound healing and inflammatory responses in conjunctival tissue. Hormonal differences may also impact eyelash growth cycles, including detachment tendencies or fiber thickness, although epidemiologic studies confirming such effects are currently lacking.

Regarding trichotillomania, clinical reports often show a female predominance (approximately 4:1), yet large‐scale population studies do not uniformly support this sex disparity [5]. A survey of around 10,000 individuals in the U.S. found nearly equal current prevalence in males (1.8%) and females (1.7%), indicating no significant sex difference [6]. Thus, while cosmetic mechanical stress and hormonal influences may explain the female dominance in our series, these remain speculative, and no direct evidence exists for sex differences in eyelash loss or subconjunctival entrapment.

The nasal predominance of eyelash embedding observed may also be related to anatomical and physiological factors. Tear drainage structures, such as the caruncle and tear puncta, lie on the nasal side of the eye, and tear flow and micro‐debris during blinking tend to move nasally. This natural trajectory may facilitate retention of detached eyelashes on the nasal conjunctiva [7].

In case two, the patient displayed mild conjunctivochalasis and a slightly deepened nasal conjunctival fornix in which the eyelash was entrapped. This finding diverges from normal anatomical data, which typically show the nasal fornix to be shallower than the temporal fornix (approx. 3.0 ± 0.9 mm vs. 5.2 ± 1.2 mm; [8]. Such atypical depth may reflect an individual anatomical variation that predisposes to eyelash entrapment.

Eyelash curvature may further contribute to the phenomenon. Tohmyoh et al. demonstrated that eyelash curvature is structurally determined by hair shaft biomechanics, resulting in a natural outward bend [9]. Procianoy et al. reported that the curvature angle increases with age, particularly in the nasal and central regions of the lower eyelid lashes [10]. These findings suggest that detached eyelashes may tend to migrate nasally due to intrinsic forces of curvature.

Taken together, tear flow direction, conjunctival fornix morphology, blinking‐induced physical forces, and eyelash curvature may interact to predispose to nasal-side subconjunctival entrapment. However, these are hypotheses; definitive anatomical or epidemiological evidence remains lacking.

Finally, as this series comprises only five cases, it lacks statistical power. Observational bias or coincidence cannot be excluded, and whether nasal entrapment reflects a true anatomical tendency or incidental occurrence remains uncertain. Accumulation of more cases and further investigation is needed to validate or refute these proposed mechanisms and to strengthen the reliability and generalizability of future findings.

Utility and safety of the extraction technique

The needle-assisted extraction technique proved to be minimally invasive under topical anesthesia, completed within a few minutes, and associated with minimal postoperative hemorrhage or complications. The primary advantage lies in its simplicity: guiding the central portion of the eyelash to the conjunctival surface with a needle under slit‑lamp visualization enables a highly controlled and safe procedure. Aslam et al. also reported a case of eyelash removal using a needle following sub‑Tenon’s anesthesia, suggesting its safety even in a postoperative setting [2]. Our technique using a 27G needle for non-invasive removal of eyelashes situated under the conjunctiva may represent a useful and minimally invasive alternative in selected cases.

Regarding safety assessment, postoperative evaluation in this case series was limited to routine clinical follow-up, during which patients were examined for obvious complications such as conjunctival hemorrhage, corneal epithelial injury, infection, or persistent inflammation. Follow-up duration was short, and no standardized or systematic complication screening protocol was employed. Therefore, while no adverse events were observed during the observation period, rare, subtle, or delayed complications cannot be excluded. The term “safe” in this report should be interpreted as referring only to the absence of clinically apparent short-term complications in this small observational series.

Clinical significance and practice recommendations

Even asymptomatic cases may provoke foreign body sensation in the patient, and there is documented risk of subsequent conjunctivitis or symblepharon formation if left untreated [11]. Given that our method can be performed promptly in an outpatient clinic without needing an operating room, it represents a practical option for routine ophthalmic practice. Additionally, the cost-effectiveness and low resource requirement further support its applicability.

Limitations and challenges

This series includes only five cases, and the follow-up period was short; thus, longer-term outcomes such as recurrence or adhesion formation remain unknown. All reported cases occurred in female patients, so potential sex differences and associations with other conditions warrant further investigation through additional case accumulation. There is also a theoretical risk of corneal or conjunctival injury with needle puncture, and comparative studies with alternative techniques, such as conjunctival incision with forceps extraction, are desirable for validation.

Although the needle-assisted extraction technique was performed safely in all cases in this series, its successful application depends on careful slit-lamp visualization, operator experience, and precise control of needle depth and direction. Therefore, this technique should be performed cautiously, particularly in eyes with severe conjunctival inflammation or poor patient cooperation.

## Conclusions

This report does not claim novelty but is intended as a descriptive account of clinical experience in an uncommon scenario. The 27‑gauge needle technique employed in this case series offers a minimally invasive, practical method for subconjunctival eyelash extraction that can be performed safely in an outpatient setting-even in asymptomatic patients. To establish its role as a standard intervention, further studies involving larger case numbers, long-term follow-up, and comparative evaluation with other techniques are necessary.

## References

[1] George S, Silvestri G (2006). Subconjuntival cilia. Eye (Lond).

[2] Aslam SA, Jayaram H, Ali N (2007). Sub-Tenon's block complicated by subconjunctival cilia. J Cataract Refract Surg.

[3] Mimura T, Nakashizuka T, Kami J, Kohmura M, Sato S, Dou K, Mori M (2011). Asymptomatic subconjunctival entrapment of a cilium. Int Ophthalmol.

[4] Aumond S, Bitton E (2018). The eyelash follicle features and anomalies: a review. J Optom.

[5] Grant JE, Dougherty DD, Chamberlain SR (2020). Prevalence, gender correlates, and co-morbidity of trichotillomania. Psychiatry Res.

[6] Thomson HA, Farhat LC, Olfson E, Levine JL, Bloch MH (2022). Prevalence and gender distribution of trichotillomania: a systematic review and meta-analysis. J Psychiatr Res.

[7] Chang AY, Purt B (2023). Biochemistry, Tear Film.

[8] Kawakita T, Kawashima M, Murat D, Tsubota K, Shimazaki J (2009). Measurement of fornix depth and area: a novel method of determining the severity of fornix shortening. Eye (Lond).

[9] Tohmyoh H, Ishihara M, Ikuta K, Watanabe T (2018). On the correlation between the curvature of the human eyelash and its geometrical features. Acta Biomater.

[10] Procianoy F, Mendonça TB, Bins CA, Lang MP (2015). Characterization of normal mediolateral angular direction of lower eyelid eyelashes in different age groups. Ophthalmic Plast Reconstr Surg.

[11] Prakash G, Sharma N, Tandon R, Titiyal JS (2010). Iatrogenic conjunctival entrapment of cilium and scleral ulceration after subtenon steroid injection. Eye Contact Lens.

